# Polarization-controlled optical holography using flat optics

**DOI:** 10.1038/s41377-020-00373-w

**Published:** 2020-07-29

**Authors:** Arka Majumdar, Shane Colburn

**Affiliations:** Electrical and Computer Engineering, University of Washington, Seattle, WA 98195 USA

**Keywords:** Sub-wavelength optics, Metamaterials

## Abstract

Due to the large number of degrees of freedom offered by nanoscale scatterers, a single flat optic can project different images at different distances depending on the polarization of the light, opening up opportunities for optical encryption and augmented reality systems.

It has long been known that images can be stored, compressed, and encrypted using optical holography^1^; however, most of these existing optical devices are bulky, and their information storage capability is often limited. With the advent of metasurfaces, it is now possible to create ultracompact optical elements using only a single surface with a near-wavelength thickness^2^. Metasurfaces are made of nanoscale scatterers, each of which can be designed to independently control the phase, amplitude, polarization, and spectrum of incident light. By precise engineering of these scatterers, it is possible to perform different functionalities with a single device, including wavelength-, angle-, and polarization-multiplexed holography. Almost all existing metasurface holograms, however, primarily rely on phase modulation.

The functionality of metasurfaces can be significantly improved if both the amplitude and phase can be manipulated together, and it was recently shown that such capability can provide high spatial-resolution metasurface holograms^3^. In that paper, the scatterers provide phase modulation via their orientation based on the geometric phase (commonly known as the Pancharatnam–Berry phase), whereas the amplitude is modulated via the size and shape of the scatterers. Such an approach could require more stringent fabrication tolerances owing to the reduced feature size. In a recent publication, Deng and coworkers developed a new way to modulate the amplitude and phase of incident light using only the orientation of the scatterers^4^. They employed a simple law of polarization, known as Malus’ law: if linearly polarized light passes through a polarizer, and the angle between the polarization axes is *θ*, the output intensity is ~cos2*θ*. Combining this with Pancharatnam–Berry phase, scatterers can be designed to independently control the phase and amplitude just by changing the orientation angle of the scatterers while keeping the size fixed. The fixed size of the scatterers can potentially make the fabrication process simpler.

Exploiting this independent control of the amplitude and phase, the authors demonstrated metasurfaces that can encode one image in the near field (information coded in the amplitude), whereas a different image can be projected in the far field (information coded in the phase) (Fig. 1). By controlling the input polarization of the light as well as the polarization orientation of the collected light, an image can be completely hidden in the far field. Such encoding of information in the polarization of light using an ultrathin metasurface can be used in many applications, including in encryption and augmented reality. For example, using polarization multiplexing, different information can be displayed in an augmented reality device in the near and far fields. In addition to providing potential applications, this work also helps us to answer a fundamental question about the information capacity of a metasurface when every degree of freedom of light is exploited.

**Fig. 1:**
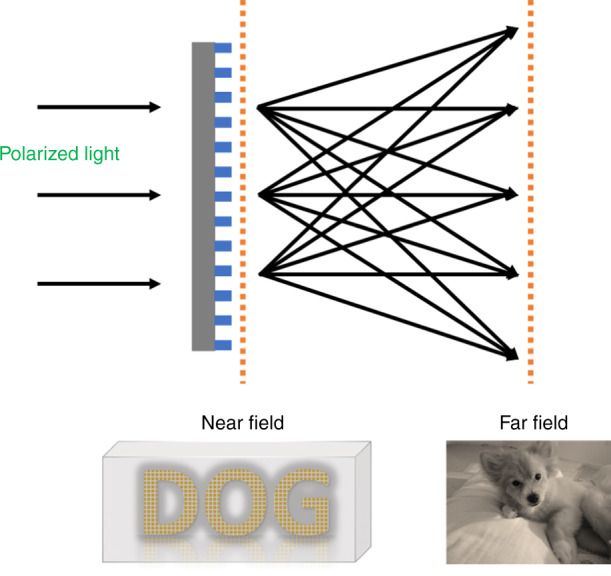
Schematic of the Metasurface. The metasurface fabricated using metasurfaces exploiting Malus’ law can provide different images in the near and far fields.

While this work primarily focuses on polarization multiplexing, the measurements still rely on multiple macroscopic optics. Cascading multiple metasurfaces could potentially reduce the overall volume of the optical system^5,6^. Finally, the metasurfaces in this work are determined via a conventional, intuition-driven design process that may be limited when exploiting multiple degrees of freedom and multiple polarization channels. Recently, reported inverse design methods^7,8^, where optimization algorithms help realize a specified functionality, could potentially circumvent such challenges.
